# Neutrophil-Lymphocyte Ratio and Mortality in the Long Life Family Study

**DOI:** 10.1093/geroni/igaa057.470

**Published:** 2020-12-16

**Authors:** Sithara Vivek, Bharat Thygarajan, Joanne M Murabito, Nicole Schupf, Joseph Zmuda, Jonas Mengel-From, Mary Wojczynski

**Affiliations:** 1 University of Minnesota, Minneapolis, Minnesota, United States; 2 Boston Medical Center, Boston, Massachusetts, United States; 3 Columbia University, New York, New York, United States; 4 University of Pittsburgh, Pittsburgh, Pennsylvania, United States; 5 University of Southern Denmark, Odense C, Denmark; 6 Washington University School of Medicine, St Louis, Missouri, United States

## Abstract

Neutrophil to Lymphocyte Ratio (NLR) reflects the balance between the innate (neutrophils) and adaptive (lymphocytes) immunity. Though NLR is a strong predictor of mortality in the general population, the distribution of NLR and its association with mortality has not been evaluated in families with exceptional longevity. Hence, we evaluated this question in the Long Life Family Study, a family based study of exceptional longevity. We used data from offspring of long lived (n=2065) family members and spousal controls (n=673). We used multivariate linear regression models adjusted for age, family relatedness, sex, field center, BMI and comorbidities (diabetes, CVD, cancer) to evaluate differences in NLR between long lived family members and spousal controls. Cox proportional hazard models were used to examine the association between NLR and mortality. 157 (7.6%) offspring in long lived families and 68 (10.1%) spousal controls were deceased during 12 years of follow up. NLR was similar among offspring in long lived families and spousal controls (1.96±1.06 vs.1.98±1.28; p=0.64). There was a significant positive association between NLR and overall mortality [HR: 1.3, 95% CI (1.01, 1.67)), p:0.04]. There was no statistically significant difference in this association among offspring in long lived families and spousal controls (p for interaction =0.16). The association between NLR and overall mortality was no longer significant [HR: 1.24; p:0.36] after adjustment for IL-6 and hsCRP. These results suggest that NLR may be a predictor of mortality in families with exceptional longevity though this association may not be independent of other inflammatory biomarkers.

